# Evaluation of thiamine as adjunctive therapy in COVID-19 critically ill patients: a two-center propensity score matched study

**DOI:** 10.1186/s13054-021-03648-9

**Published:** 2021-06-30

**Authors:** Khalid Al Sulaiman, Ohoud Aljuhani, Maram Al Dossari, Asma Alshahrani, Aisha Alharbi, Rahmah Algarni, Majed Al Jeraisy, Shmeylan Al Harbi, Abdulmalik Al Katheri, Fahad Al Eidan, Abdulkareem M. Al Bekairy, Nouf Al Qahtani, Mashael Al Muqrin, Ramesh Vishwakarma, Ghassan Al Ghamdi

**Affiliations:** 1 Pharmaceutical Care Department, King Abdulaziz Medical City (KAMC)/King Abdullah International Medical Research Center (KAIMRC), Riyadh, Saudi Arabia; 2 Department of Pharmacy Practice, Faculty of Pharmacy, King Abdulaziz University, Jeddah, Saudi Arabia; 3 Department of Pharmacy Practice, Faculty of Pharmacy, King Khalid University, Abha, Saudi Arabia; 4 Pharmaceutical Care Department, King Abdulaziz University Hospital, Jeddah, Saudi Arabia; 5 College of Medicine, King Saud Bin Abdulaziz University for Health Sciences, Riyadh, Saudi Arabia; 6 Intensive Care Department, King Abdulaziz Medical City, Riyadh, Saudi Arabia; 7 College of Pharmacy, King Saud Bin Abdulaziz University for Health Sciences, Riyadh, Saudi Arabia; 8 Biostatistics and Bioinformatics Department, King Abdullah International Medical Research Center, Riyadh, Saudi Arabia

**Keywords:** COVID-19, SARS-CoV-2, Thiamine, Vitamin B1, Vitamins, Critically ill, Intensive care units (ICUs), 30-day mortality

## Abstract

**Background:**

Thiamine is a precursor of the essential coenzyme thiamine pyrophosphate required for glucose metabolism; it improves the immune system function and has shown to reduce the risk of several diseases. The role of thiamine in critically ill septic patient has been addressed in multiple studies; however, it’s role in COVID-19 patients is still unclear. The aim of this study was to evaluate the use of thiamine as an adjunctive therapy on mortality in COVID-19 critically ill patients.

**Methods:**

This is a two-center, non-interventional, retrospective cohort study for critically ill patients admitted to intensive care units (ICUs) with a confirmed diagnosis of COVID19. All patients aged 18 years or older admitted to ICUs between March 1, 2020, and December 31, 2020, with positive PCR COVID-19 were eligible for inclusion. We investigated thiamine use as an adjunctive therapy on the clinical outcomes in critically ill COVID-19 patients after propensity score matching.

**Results:**

A total of 738 critically ill patients with COVID-19 who had been admitted to ICUs were included in the study. Among 166 patients matched using the propensity score method, 83 had received thiamine as adjunctive therapy. There was significant association between thiamine use with in-hospital mortality (OR = 0.39; 95% CI 0.19–0.78; *P* value = 0.008) as well as the 30-day mortality (OR = 0.37; 95% CI 0.18–0.78; *P* value = 0.009). Moreover, patients who received thiamine as an adjunctive therapy were less likely to have thrombosis during ICU stay [OR (95% CI) 0.19 (0.04–0.88), *P* value = 0.03].

**Conclusion:**

Thiamine use as adjunctive therapy may have potential survival benefits in critically ill patients with COVID-19. Additionally, it was associated with a lower incidence of thrombosis. Further interventional studies are required to confirm these findings.

**Supplementary Information:**

The online version contains supplementary material available at 10.1186/s13054-021-03648-9.

## Introduction

With the rapid spread of the disease, as well as the high mortality rates among critically ill patients, there are many studies with different methodology approaches conducted among COVID-19 patients to investigate the effectiveness of many medications (e.g., steroids, antivirals, immunomodulators) and respiratory support strategies (e.g., prone position, volume protected strategy) [1–3]. Several proposed vitamins and trace elements therapies are currently under investigation, such as ascorbic acid and zinc [4–7]. However, the role of other vitamins, especially thiamine in COVID-19 critically ill patients, is still unclear.

Thiamine is a precursor of the essential coenzyme thiamine pyrophosphate (TPP) required for glucose metabolism; it improves the immune system function and has been shown to reduce the risk of several diseases [8]. Besides that, thiamine diphosphate (TDP)-dependent enzymes are a vast group of proteins that contribute to many catabolism reactions of enzymes, along with neurotransmitters biosynthesis and antioxidant activity [9]. Thiamine deficiency is a serious medical condition that may lead to many complications requiring medical interventions such as Wernicke's encephalopathy, delirium, and beriberi [10–12].

The role of thiamine in COVID-19 patients is still unclear; however, its role in critically ill patients has been addressed in multiple studies [13–15]. A randomized controlled trial by Moskowitz et al. shows that thiamine was associated with significantly lower lactate levels, serum creatinine and a possible decrease in 30-day mortality [13]. In addition, another observational study shows that thiamine administration within 24 hours  was associated with an improved possibility of lactic acid clearance and reduced 28-day mortality in critically ill patients with septic shock [14].

The question remains about the thiamine role in COVID-19 critically ill patients. Antibodies and, importantly, T-cells are required to eliminate the SARS-CoV-2 virus; thiamine deficiency can potentially result in inadequate antibody responses and more severe symptoms [8]. Thiamine also works as a carbonic anhydrase isoenzyme inhibitor; thus, high doses of thiamine given to patients at the early stages of COVID-19 could limit hypoxia and decrease hospitalization [16]. Additionally, an in-vitro study found that high-dose thiamine lowers the T-helper cells (Th-17) cell pro-inflammatory response believed to be associated with the COVID-19 cytokine storm [17].

As of July 15, 2020, over 300 COVID-19 patients were treated with a protocol named MATH+ protocol which combines a range of substances: methylprednisolone, ascorbic acid, thiamine, heparin and several additional components, including melatonin, zinc, vitamin D, atorvastatin and famotidine [4]. Unfortunately, no current studies specifically investigate thiamine's effect in COVID-19 patients to the best of our knowledge. Therefore, our study aims to determine the association between thiamine use as an adjunctive therapy and the clinical outcomes in COVID-19 critically ill patients.

## Methods

### Study design

A retrospective study of critically ill patients admitted to intensive care units (ICUs) with a confirmed diagnosis of COVID-19 in two-tertiary care centers in Saudi Arabia between March 1 and December 31, 2020. All patients who met the inclusion criteria during the study period were included. Eligible patients have been divided into two groups based on thiamine use as adjunctive therapy during ICU stay; there were no pre-defined criteria at the two centers for thiamine initiation. Intravenous (IV) or enteral) thiamine was given empirically and not based on baseline thiamine levels. Patients were observed during their hospital stay until discharge or in-hospital death, whichever occurred first. The study was approved by the Ministry of National Guard Health Affairs-Institutional Review Board (IRB), Riyadh, Saudi Arabia (Study Number: RC20/589/R).

### Participants

Patients who were 18 years of age or older and admitted to ICU with confirmed COVID-19 by reverse transcriptase-polymerase chain reaction (RT-PCR) on nasopharyngeal or throat swabs were eligible for inclusion. Patients were excluded if the ICU length of stay (LOS) was less than a day or labeled as "Do-Not-Resuscitate" code status within 24 hours of ICU admission.

### Setting

This study was conducted in two large, tertiary governmental hospitals; King Abdulaziz Medical City, Riyadh (KAMC-RD) and King Abdulaziz University Hospital, Jeddah (KAUH-JD). The ICUs admit medical, surgical, trauma, burn and transplant patients and operate as a closed unit with 24/7 on-site coverage by critical care board-certified intensivists. The distributions of total enrolled patients were 77% and 23% in KAMC-CR and KAUH-JD, respectively. The primary site was KAMC-RD.

### Data collection

Data gathered from the patients' electronic medical records included demographic data (see Additional file 1), thiamine use, acute physiology and chronic health evaluation II (APACHE II), sequential organ failure assessment (SOFA) and nutrition risk in critically ill (NUTRIC). Comorbidities, vital signs, laboratory tests, the needs for mechanical ventilation (MV), MV parameters (e.g., PaO_2_/FiO_2_ ratio, FiO_2_ requirement) and inflammatory markers (C-reactive protein (CRP), procalcitonin) within 24 hours  of ICU admission were recorded. Additionally, ICU complication (s) during ICU stay (e.g., Acute Kidney Injury (AKI), thrombosis/infarction), ICU length of stay (LOS), hospital LOS, mechanical ventilation (MV) duration and ICU/in-hospital mortality were collected for eligible patients.

### Outcomes

The primary endpoints were determining the association between using thiamine as adjunctive therapy with the in-hospital and 30-day mortality in critically ill patients with COVID-19. The secondary endpoints include evaluation of MV duration, length of stay and complication (s) during ICU stay (i.e., acute kidney injury, acute liver injury, respiratory failure and thrombosis/ infarction).

### Definition(s)

- The acute kidney injury was defined using the AKIN definition [18].
- Thrombosis/ infarction was defined using ICD10-CM code (i.e., Myocardial infarction (MI), ischemic stroke, pulmonary embolism, deep vein thrombosis) [6].
- Respiratory failure was defined as either hypoxemic respiratory failure (PaO_2_ < 60 mm Hg with a normal or low arterial carbon dioxide tension (PaCO_2_) or hypercapnic respiratory failure (PaCO_2_ > 50 mm Hg) that requires mechanical ventilation [16].
- Acute liver injury, defined as alanine aminotransferase (ALT) exceeds three times the normal upper limit (ULN) or doubled in patients with elevated baseline ALT [6].

### Data management and statistical analysis

Categorical variables were reported using numbers and percentages, whereas continuous variables reported using means with standard deviation (SD) or medians with interquartile range (IQR) when appropriate. The normality assumptions were assessed for all numerical variables using a statistical test (i.e., Shapiro–Wilk test) and graphical representation (i.e., histograms and Q–Q plots). We compared categorical variables using the Chi-square or Fisher exact test, normally distributed numerical variables with the Mann–Whitney U test.

Model fit was assessed using the Hosmer–Lemeshow goodness-of-fit test. Multivariable logistic regression and negative binomial regression were used to determine the relationship between thiamine use and different outcomes considered in this study. The odds ratios (OR) and estimates with the 95% confidence intervals (CI) were reported for the associations. No imputation was made for missing data as the cohort of patients in our study was not derived from random selection.

Propensity score matching procedures (Proc PS match) (SAS, Cary, NC) were used to match patients who received thiamine with patients who did not based on  patient’s baseline severity scores (i.e., APACHE II, SOFA, and NUTRIC scores), systemic use of corticosteroids and study centers. A greedy nearest neighbor matching method was used in which one patient in the control group was matched with each patient in the thiamine (treated) group. This eventually produces the smallest within-pair difference among all available pairs with treated patients. These patients were matched only if the difference in the logits of the propensity scores for pairs of patients from the two groups was less than or equal to 0.5 times the pooled estimate of the standard deviation. We considered a *P* value of < 0.05 statistically significant and used SAS version 9.4 for all statistical analyses.

## Results

A total of 738 critically ill patients with COVID-19 admitted to ICUs at the two governmental hospitals were included in the study. Thiamine was given to 88 patients, whereas 650 patients did not receive thiamine. A total of 166 patients were included after propensity score matching had been conducted using baseline severity scores, systemic use of corticosteroids and study centers. The median (Q1, Q3) dose of thiamine given per day was 100 mg (50, 200) with a median duration of seven days. The majority of patients received thiamine by intravenous administration (57%).

### Demographic and clinical characteristics

Among critically ill patients admitted to ICUs, the patients' average age was 60 years (± 15). A total of 531 (72%) patients were male (Table 1, Additional file 1). Diabetes mellitus (61%) was the most common coexisting illness, followed by hypertension (56.8%) and dyslipidemia (23.2%). Coexisting illness between the two groups was not statistically significant (Table 2, Additional file 2).

**Table 1:** Regression analysis for the outcomes

Outcomes	Before propensity score					After propensity score				
	Control (650 patients)	Thiamine (88 patients)	*P *value	Odds Ratio (OR) (95% CI)	*P *value	Control (83 patients)	Thiamine (83 patients)	*P *value	Odds ratio (OR) (95% CI)	*P *value
30-day mortality, n (%)^#^	264 (40.6)	18 (20.5)	0.002^^	0.45 (0.24,0.84)	0.01*^	30 (36.1)	15 (18.1)	0.009^^	0.37 (0.18,0.78)	0.009^$^
In-hospital mortality, n (%)^#^	313 (48.2)	22 (25)	0.009^^	0.37 (0.18,0.78)	0.009*^	35 (42.2)	19 (22.9)	0.008^^	0.39 (0.19,0.78)	0.008^$^

				Beta coefficient (95% CI)	*P* value				Beta coefficient (95% CI)	*P* value
MV duration during ICU, Median (Q1, Q3) ^&#^	10.0 (4.0, 17.0)	7.0 (3.0, 16.0)	0.17^	0.02 (− 0.24, 0.30)	0.84*^	11.0 (5.0, 20.0)	7.0 (3.0, 20.0)	0.68^	− 0.13 (− 0.54, 0.27)	0.51^$^^*^
ICU Length of Stay (Days), Median (Q1, Q3) ^&^	8.0 (5.0, 14.0)	8.0 (5.0,13.0)	0.89^	0.07 (− 0.12, 0.27)	0.49*^	8.0 (4.0, 12.0)	8.0 (5.0, 13.0)	0.78^	0.11 (− 0.19, 0.39)	0.48^$^^*^
Hospital Length of Stay (Days), Median (Q1, Q3)^&^	17.0 (11.0, 26.5)	15.0 (10.0, 24.5)	0.47^	0.04 (− 0.12,0.21)	0.59*^	13.0 (11.0, 21.0)	15.0 (10.0, 25.0)	0.30^	0.08 (− 0.17,0.33)	0.52^$^^*^

^#^Denominator of the percentage is the total number of patients

^Wilcoxon rank sum test is used to calculate the *P* value

^^Chi-square test is used to calculate the *P* value

^$^*Propensity score adjusted negative binomial regression is used to calculate the beta coefficient (estimates) and *P* value

^$^Propensity score adjusted logistic regression is used to calculate odds ratio and *P *value

^*^^Multivariable logistic regression is used after adjusting for patient’s baseline severity scores, systemic use of corticosteroids and study centers to calculate odds ratio and *P* value

^&^Denominator is patients who survived

^&#^Denominator is patients who have respiratory failure requiring MV during ICU stay

**Table 2:** Regression analysis for ICU complication(s) during ICU stay

Outcomes	Before propensity score					After propensity score				
	Control (650 patients)	Thiamine (88 patients)	*P* value	Odds ratio (OR) (95%CI)	*P* value	Control (83 patients)	Thiamine (83 patients)	*P* valve	Odds ratio (OR) (95%CI)	*P* value
Acute kidney injury (AKI), n (%)#	304/639 (47.6)	31/87 (35.6)	0.04^^	0.56 (0.36,0.86)	0.009^*^^	21/82 (43.9)	15/83 (33.7)	0.26^^	0.91 (0.49,1.68)	0.75^$^
Liver injury, n (%)#	70/637(10.9)	7/87 (8.1)	0.40^^	0.09 (0.04,0.19)	< .0001^*^^	5/82 (6.1)	6/83 (7.2)	0.77^^	0.93 (0.30,2.87)	0.90^$^
Respiratory failure required MV, n (%)$*	67/197 (34.0)	16/42 (38)	0.61^^	1.05 (0.49, 2.22)	0.90*^	14/31 (45.1)	16/40 (40)	0.66^^	0.95 (0.35, 2.57)	0.92^$^
Thrombosis/infarction during ICU, n(%)#	71/632 (11.2)	2/87 (2.3)	0.01^^	0.03 (0.008,0.10)	< .0001^*^^	9/81 (11.1)	2/83 (2.4)	0.02^^	0.19 (0.04,0.88)	0.03^$^

^#^Denominator of the percentage is the total number of patients

^$^*Denominator of the percentage is non-mechanically ventilated patients with 24 h of ICU admission

^^Chi-square test is used to calculate the *P* value

^$^Propensity score adjusted logistic regression is used to calculate odds ratio and *P* value

*^Multivariable logistic regression is used after adjusting for patient’s baseline severity scores, systemic use of corticosteroids and hospital center to calculate odds ratio and *P* value

The baseline severity scores (i.e., APACHE II, SOFA and NUTRIC scores), mechanical ventilation (MV) needs within 24 h of ICU admission, and laboratory tests (i.e., INR, Fibrinogen, CRP, Ferritin, HCT, pH) were significantly higher among patients who did not receive thiamine during ICU stay. On the other hand, systematic corticosteroids use during ICU stay, and phosphorus levels were higher in the thiamine group. However, after conducting propensity score matching, most of these baseline and demographic characteristics were similar between the two groups (Table 1, Additional file 1).

### Study outcomes

**Fig. 1:**
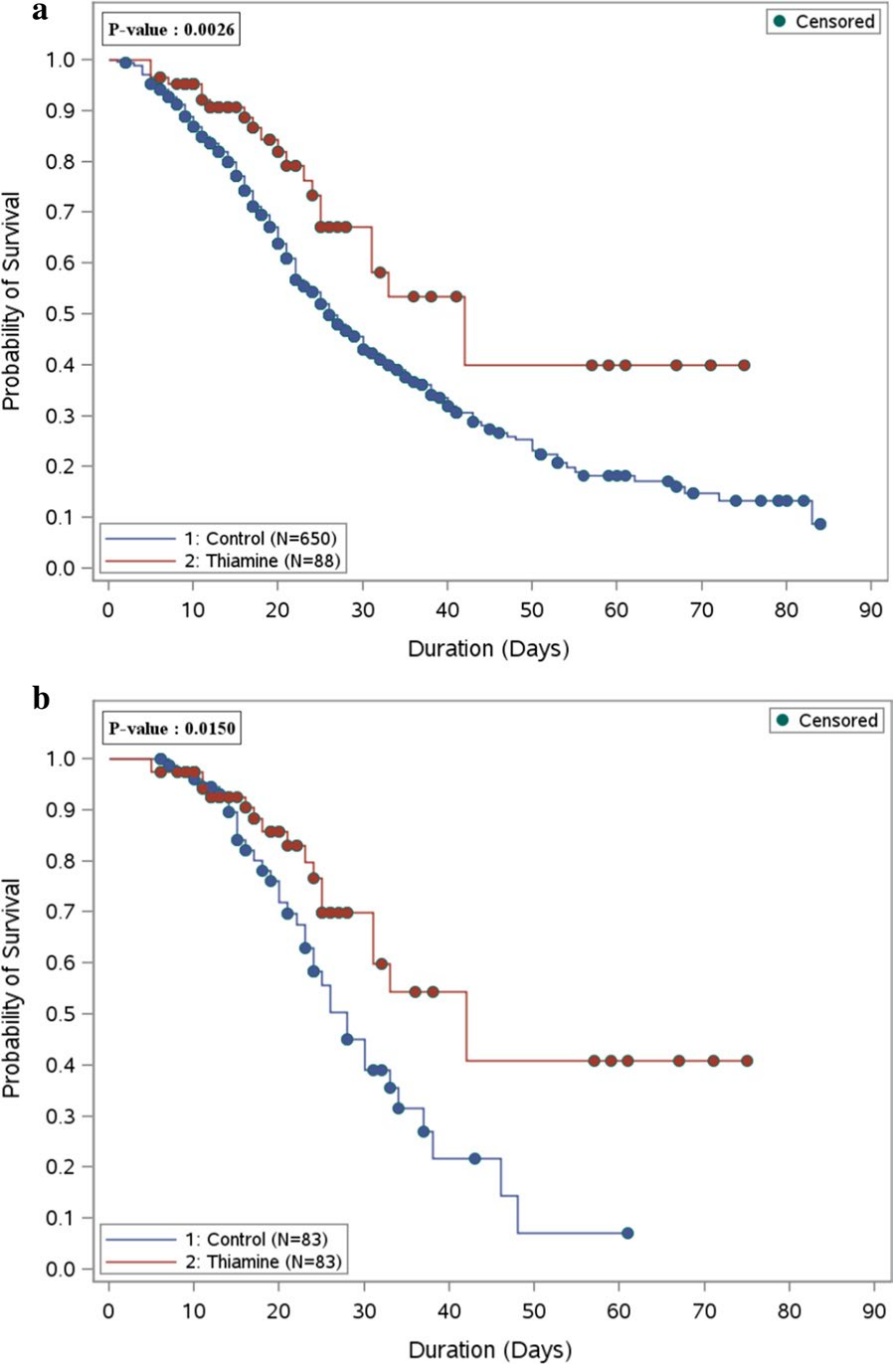
**a** Overall survival plot during the hospital stay comparing patients who received thiamine (88 patients) as adjunctive therapy versus the control group (650 patients)—before PS matching. **b** Overall survival plot during the hospital stay comparing patients who received thiamine (83 patients) as adjunctive therapy versus the control group (83 patients)—after PS matching

There were fifteen patients (18.1%) who died during ICU stay among the thiamine group, compared with thirty patients (36.1%) in the other group. In other words, patients who received thiamine as adjunctive therapy during ICU stay had a lower 30-day mortality rate by 63% [OR (95% CI): 0.37 (0.18, 0.78), *P* value = 0.009] (Table 1). Additionally, thiamine use was associated significantly with a lower in-hospital mortality rate by 61% [OR (95% CI): 0.39 (0.19,0.78), *P* value = 0.008]. The overall survival probabilities were higher during hospital stay among patients who received thiamine before and after propensity score-matched (Fig. 1a, b).

The duration of mechanical ventilation (Beta estimate − 0.13 CI − 0.54, 0.27; *P* value = 0.51), ICU length of stay (LOS) (Beta estimate 0.11 CI − 0.19, 0.39; *P* value = 0.48) and hospital LOS (Beta estimate 0.08 CI − 0.17, 0.33; *P* value = 0.52) did not differ significantly in patients who received thiamine as adjunctive therapy compared to patients who did not (Table 1).

### Complications during ICU stay

Critically ill patients who received thiamine as an adjunctive therapy were less likely to have thrombosis during ICU stay by 81% [OR (95% CI) 0.19 (0.04, 0.88), *P* value = 0.03] (Table 2). Moreover, acute kidney injury [OR (95% CI) 0.91 (0.49, 1.68)] and liver Injury [OR (95% CI) 0.93 (0.30, 2.87)] were lower in the thiamine group by 9% and 7%, respectively. However, the differences were not statistically significant (Table 2).

## Discussion

Our study is a two-center, non-interventional retrospective study of critically ill patients admitted to ICUs with a confirmed diagnosis of COVID-19. It investigates the correlation between thiamine use as adjunctive therapy and the clinical outcomes. The 30-day mortality rate was significantly lower in the thiamine group with a *P* value of (*P* = 0.009) after using propensity score matching that takes into consideration the use of the corticosteroid since it showed survival benefits in the recovery trial [26].

Our findings agree with a substantial number of studies published over the past decades and reported a survival benefit with thiamine in non-COVID19 critically ill patients. However, there are no current trials that directly investigate the effect of thiamine in critically ill COVID-19 patients. Woolum et al. proved the relation between early thiamine administration to critically ill patients with septic shock during the first 24 hours  of admission with rapid lactate clearance and decreased 28-day mortality rates [14]. However, the recent VITAMINS trial tested the HAT protocol (hydrocortisone, ascorbic acid and thiamine) in critically ill patients with septic shock and found no survival benefit [21].

Severe COVID-19-infected patients may develop malnutrition and risk for refeeding syndrome due to low food intake prior to ICU admission [22]. Thiamine deficiency plays a major role in malnutrition in critically ill patients, which leads to the inability to create adenosine triphosphate (ATP), inability to use oxygen, high-output cardiac failure, cardiovascular collapse and death when untreated [23]. One retrospective observational study conducted in Wuhan found that critically ill COVID-19 patients with higher Nutritional Risk Screening 2002 (NRS) had a higher risk of mortality and longer hospital stay [22]. Preadmission nutritional status in our patients has been assessed using NUTRIC score due to the unavailability of NRS-2002-related information (e.g., weight loss history, food intake history prior to ICU admission). Moreover, phosphorus levels were evaluated in our cohort for further nutrition assessment, the mean phosphorus level was 1.05 (0.36) mmol/L and 1.16 (0.38) mmol/L in control and thiamine group (*P* = 0.07), respectively. The preadmission low nutrition status due to COVID-19 infection combined with the known low intake of some micronutrients (such as zinc and selenium) in the Saudi population might explain the benefits of thiamine observed in our study [11, 24].

The ICU length of stay was not statistically significant between the groups (*P-*value = 0.48), with a median duration of eight days. A lack of studies assessed the thiamine's impact on ICU LOS in COVID-19 critically ill patients. Conversely, a retrospective study by Mitchell et al. investigates the benefits of thiamine, vitamin C, and hydrocortisone as a combination in septic patients and found a significant difference in the ICU LOS [25].

In our data, the incidence of AKI was lower in the thiamine group, which may explain a kidney protective effect but was not statistically significant. Acute kidney injury is one of the most frequent complications during critical illness and could contribute to a malnutrition state and impaired patients' immunity [27]. A randomized, double-blind, placebo-controlled trial found that serum creatinine levels were lower in patients who received a high dose of thiamine and were less likely to have kidney failure requiring renal replacement therapy (RRT) [13].

Interestingly, thiamine use was found to be associated with a statistically significant reduction in thrombosis by 81% compared to the control group [OR (95% CI) 0.19 (0.040, 0.884), *P* value = 0.03]. The exact mechanism for thrombosis reduction observed in our cohort with thiamine use is unknown. This finding worth further investigation and should trigger future research to address the precise impact of thiamine on the prevention of thrombosis in COVID-19 critically ill patients.

Our study's uniqueness lies in the lack of extensive well-conducted studies connecting thiamine administration's effect directly to a positive impact on mortality rates in COVID 19 critically ill patients. However, the study may have been affected by several limitations, including our design observational nature, and some residual confounding factors are still possible. Therefore, we conducted several analyses to control these variables using multivariable regression adjustment after propensity score matching. Additionally, thiamine initiation in our centers was primarily based on clinical judgment; thus, treating physicians' bias toward using one treatment regimen versus another cannot be ruled out. Also, thiamine levels were not measured for patients neither initially on admission nor during ICU stay.

Thiamine has a good safety profile, and readily available at low cost; therefore, it can be considered as a part of COVID-19 critically ill patients' therapeutic management protocols. Further interventional studies are required to confirm our findings.

## Conclusion

Thiamine use as adjunctive therapy may have potential survival benefits in critically ill patients with COVID-19. Additionally, it was associated with a lower incidence of thrombosis. Systemic thiamine administration could be considered as a part of COVID-19 management upon ICU admission.

## Supplementary Information

**Additional file 1: Table 1**. Summary of demography and baseline characteristics

**Additional file 2: Table 2**. Co-existing illness

## Data Availability

The datasets used and/or analyzed during the current study are available from the corresponding author on reasonable request.

## Abbreviations

ICUs: Intensive care units
COVID-19: Coronavirus disease
MV: Mechanical ventilation
Vitamin B1: Thiamine
LOS: Length of stay
APACHE II: Acute physiology and chronic health evaluation II
SOFA: Sequential organ failure assessment
NUTRIC: Nutrition risk in the critically ill
