# Supplementary material for: Evaluation of thiamine as adjunctive therapy in COVID-19 critically ill patients: a two-center propensity score matched study
Source: Crit Care. 2021 Jun 30;25:223. doi: 10.1186/s13054-021-03648-9 (PMC8242279; doi:10.1186/s13054-021-03648-9)
Supplement: Supplementary file 1 — Additional file 1: Table 1. Summary of demography and baseline characteristics [file 13054_2021_3648_MOESM1_ESM.docx]

Table e1, Supplementary material, Baseline characteristics

|  | **Before Propensity Score Matching** | | | | **After Propensity Score Matching** | | | |
| --- | --- | --- | --- | --- | --- | --- | --- | --- |
| **Variables** | **Overall (738)** | **Control** | **Thiamine** | **P-value** | **Overall (166)** | **Control** | **Thiamine** | **P-value** |
|  |  | **(N=650)** | **(N=88)** |  |  | **(N=83)** | **(N=83)** |  |
| **Age (Years), Mean (SD)** | 60.7 (14.81) | 60.7 (14.67) | 60.6 (15.91) | 0.984* | 60.0 (16.00) | 60.0 (15.96) | 60.16 (16.13) | 0.877* |
| **Gender – Male, n (%)** | 531 ( 72.0 ) | 466 ( 71.7 ) | 65 ( 73.9 ) | 0.670^^ | 120 ( 72.3 ) | 57 ( 68.7 ) | 63 ( 75.9 ) | 0.298^^ |
| **Weight (kg), Mean (SD)** | 81.1 (18.92) | 81.0 (18.75) | 81.5 (20.17) | 0.956^ | 80.9 (19.30) | 81.1 (18.57) | 80.8 (20.11) | 0.481^ |
| **Height (cm), Mean (SD)** | 163.5 (11.36) | 163.6 (11.23) | 163.7 (12.34) | 0.517^ | 163.3 (10.95) | 162.1 (9.09) | 163.7 (12.57) | 0.342^ |
| **Body mass index (BMI), Mean (SD)** | 30.3 (7.26) | 30.3 (7.20) | 30.2 (7.68) | 0.929^ | 30.3 (7.30) | 30.6 (6.91) | 29.9 (7.71) | 0.373^ |
| **APACHE II score, Median (IQR)** | 12.0 (8.00, 22.00) | 13.0 (8.00, 23.00) | 10.5 (7.00, 16.00) | 0.001^ | 10.0 (7.00, 14.00) | 10.0 (7.00, 14.00) | 10.0 (6.00, 14.00) | 0.952^ |
| **SOFA score , Median (IQR)** | 5.0 (3.00, 8.00) | 5.0 (3.00, 8.00) | 4.0 (2.00, 6.00) | 0.001^ | 4.0 (2.00, 6.00) | 4.0 (2.00, 6.00) | 4.0 (2.00, 6.00) | 0.357^ |
| **NUTRIC Score, Median (IQR)** | 3.0 (2.00, 6.00) | 4.0 (2.00, 6.00) | 3.0 (2.00, 4.00) | 0.001^ | 3.0 (2.00, 4.00) | 3.0 (2.00, 4.00) | 3.0 (1.00, 4.00) | 0.617^ |
| **Systemic Corticosteroids during ICU, n (%)** | 639 ( 87.9 ) | 556 ( 86.9 ) | 83 ( 95.4 ) | 0.022^^ | 160 ( 96.4 ) | 81 ( 97.6 ) | 79 ( 95.2 ) | 0.681** |
| **Glasgow Coma Scale (GCS) Baseline, Median (IQR)** | 15.0 (11.00, 15.00) | 15.0 (10.00, 15.00) | 15.0 (15.00, 15.00) | 0.001^ | 15.0 (15.00, 15.00) | 15.0 (15.00, 15.00) | 15.0 (15.00, 15.00) | 0.306^ |
| **Serum Creatinine (μmol/L), Median (IQR)** | 91.0 (71.00, 137.00) | 93.0 (71.00, 138.00) | 78.5 (66.50, 127.00) | 0.027^ | 81.0 (65.00, 121.00) | 84.0 (63.00, 122.00) | 77.0 (66.00, 113.00) | 0.788^ |
| **Acute Kidney Injury (AKI) within 24 hours of ICU admission, n (%)** | 211 ( 28.9 ) | 193 ( 30.0 ) | 18 ( 20.5 ) | 0.063^^ | 37 ( 22.3 ) | 22 ( 26.5 ) | 15 ( 18.1) | 0.191^^ |
| **MV during ICU stay within 24 hours, n (%)** | 489 ( 66.9 ) | 443 ( 68.9 ) | 46 ( 52.3 ) | 0.001^^ | 94 ( 56.6 ) | 51 ( 61.4 ) | 43 ( 51.8 ) | 0.210^^ |
| **Blood sugar level (mmol/L) Baseline, Median (IQR)** | 11.7 (7.95, 16.75) | 11.7 (7.90, 16.90) | 12.0 (8.70, 15.20) | 0.604^ | 11.9 (7.80, 15.20) | 11.9 (7.90, 15.80) | 11.9 (7.80, 14.80) | 0.931^ |
| **Lactic acid Baseline (mmol/L), Median (IQR)** | 1.8 (1.30, 2.50) | 1.8 (1.31, 2.54) | 1.7 (1.29, 2.18) | 0.167^ | 1.7 (1.30, 2.26) | 1.7 (1.32, 2.55) | 1.7 (1.29, 2.19) | 0.608^ |
| **Platelets count Baseline (10^9/L), Median (IQR)** | 251.0 (191.00, 328.00) | 252.00 (190.00, 324.00) | 249.0 (199.50, 341.50) | 0.738^ | 250.0 (193.00, 334.00) | 248.0 (188.00, 328.00) | 250.0 (193.00, 347.00) | 0.569^ |
| **Total WBC Baseline (10^9/L), Median (IQR)** | 10.1 (7.00, 14.10) | 10.1 (7.12, 14.40) | 9.3 (6.27, 13.00) | 0.248^ | 9.4 (6.46, 13.20) | 9.6 (6.76, 13.00) | 9.4 (6.27, 13.20) | 0.920^ |
| **International normalized ratio (INR), Median (IQR)** | 1.1 (1.04, 1.19) | 1.1 (1.04, 1.20) | 1.1 (1.01, 1.14) | 0.002^ | 1.1 (1.02, 1.15) | 1.1 (1.04, 1.15) | 1.1 (1.01, 1.14) | 0.118^ |
| **Activated partial thromboplastin time (aPTT) Baseline (Seconds), Median (IQR)** | 29.7 (26.80, 33.25) | 30.0 (26.65, 33.30) | 28.3 (26.80, 31.65) | 0.087^ | 29.0 (27.00, 31.70) | 29.3 (27.00, 32.70) | 28.2 (26.80, 31.50) | 0.339^ |
| **Total bilirubin (μmol/L), Median (IQR)** | 10.0 (7.00, 14.60) | 10.0 (7.00, 14.30) | 10.0 (6.70, 15.65) | 0.867^ | 9.9 (6.90, 14.70) | 8.9 (6.70, 14.00) | 10.1 (7.00, 15.90) | 0.349^ |
| **Alanine aminotransferase (ALT) Baseline (U\L), Median (IQR)** | 38.0 (25.00, 66.00) | 38.5 (25.00, 66.00) | 37.5 (24.50, 60.50) | 0.616^ | 37.0 (25.00, 66.00) | 35.5 (24.00, 66.00) | 38.0 (25.00, 66.00) | 0.650^ |
| **Aspartate aminotransferase (AST) Baseline (U\L), Median (IQR)** | 55.0 (35.00, 80.00) | 55.0 (36.00, 82.00) | 53.0 (33.00, 68.00) | 0.249^ | 53.0 (33.00, 69.00) | 53.0 (36.50, 71.00) | 52.5 (33.00, 68.00) | 0.784^ |
| **Albumin Baseline (gm/L), Median (IQR)** | 33.0 (30.00, 36.30) | 33.0 (29.00, 36.60) | 33.0 (31.00, 36.00) | 0.903^ | 33.0 (31.00, 36.00) | 33.0 (30.00, 36.00) | 33.0 (31.00, 36.00) | 0.764^ |
| **Blood urea nitrogen (BUN) Baseline (mmol/L), Median (IQR)** | 7.2 (4.90, 12.30) | 7.3 (5.00, 12.60) | 6.8 (4.70, 11.80) | 0.357^ | 6.9 (4.60, 10.40) | 7.4 (4.30, 9.90) | 6.7 (4.70, 10.40) | 0.743^ |
| **Creatine phosphokinase (CPK) (U/l), Median (IQR)** | 200.5 (78.00, 514.00) | 206.0 (78.00, 558.00) | 171.0 (69.00, 359.00) | 0.123^ | 153.0 (67.00, 308.00) | 153.0 (67.00, 308.00) | 161.5 (69.00, 292.00) | 0.952^ |
| **C-reactive protein (CRP) (mg/l), Median (IQR)** | 156.0 (86.00, 224.00) | 159.0 (92.00, 230.00) | 133.0 (73.00, 170.00) | 0.019^ | 138.0 (73.00, 205.00) | 156.0 (77.00, 251.00) | 130.0 (72.00, 170.00) | 0.107^ |
| **Procalcitonin (ng/ml), Median (IQR)** | 0.35 (0.15, 1.38) | 0.38 (0.16, 1.45) | 0.2 (0.08, 0.64) | 0.018^ | 0.3 (0.12, 0.67) | 0.3 (0.16, 0.85) | 0.2 (0.08, 0.64) | 0.208^ |
| **Ferritin (ug/l), Median (IQR)** | 856.1 (414.20, 2026.40) | 906.8 (433.40, 2078.80) | 734.6 (350.80, 1390.50) | 0.014^ | 743.4 (406.60, 1567.60) | 728.5 (494.00, 1774.00) | 778.2 (353.90, 1417.90) | 0.346^ |
| **Fibrinogen Level (gm/l), Median (IQR)** | 6.7 (4.93, 332.00) | 6.9 (5.01, 399.00) | 5.4 (3.89, 6.93) | 0.001^ | 5.7 (3.90, 6.93) | 5.8 (4.36, 7.18) | 5.4 (3.88, 6.93) | 0.615* |
| **Bicarbonate (CO2) Baseline (mmol/L), Median (IQR)** | 21.0 (19.00, 24.00) | 21.0 (19.00, 24.00) | 21.0 (19.00, 24.00) | 0.709^ | 21.5 (19.00, 24.00) | 22.0 (19.00, 24.00) | 21.0 (19.00, 24.00) | 0.690^ |
| **Chloride (CL) Baseline (mmol/L), Median (IQR)** | 102.0 (99.00, 106.00) | 102.0 (99.00, 106.00) | 102.0 (99.00, 106.25) | 0.835^ | 103.0 (100.00, 106.00) | 103.0 (100.00, 106.00) | 102.0 (99.00, 106.00) | 0.593^ |
| **Potassium (K) Baseline (mmol/L), Median (IQR)** | 4.2 (3.75, 4.75) | 4.2 (3.70, 4.78) | 4.2 (3.90, 4.60) | 0.635^ | 4.2 (3.80, 4.60) | 4.1 (3.70, 4.60) | 4.2 (3.90, 4.60) | 0.243^ |
| **Phosphorus level Baseline (mmol/L), Mean (SD)** | 1.07 (0.39) | 1.05 (0.39) | 1.16 (0.39) | 0.02^ | 1.10 (0.38) | 1.05 (0.36) | 1.16 (0.38) | 0.07 |
| **Sodium baseline (mmol/L), Median (IQR)** | 137.0 (134.75, 140.75) | 137.0 (134.60, 141.00) | 137.0 (135.00, 140.00) | 0.933^ | 137.5 (135.00, 140.00) | 138.0 (135.00, 140.00) | 137.0 (135.00, 140.00) | 0.606^ |
| **Hematocrit (Hct) (L\L), Median (IQR)** | 0.4 (0.35, 0.47) | 0.4 (0.35, 0.48) | 0.4 (0.35, 0.43) | 0.046^ | 0.4 (0.35, 0.43) | 0.4 (0.35, 0.43) | 0.4 (0.36, 0.43) | 0.618^ |
| **Calculated serum osmolality, Median (IQR)** | 287.1 (279.17, 295.30) | 286.4 (278.57, 294.95) | 289.5 (283.70, 298.80) | 0.018^ | 290.7 (283.70, 297.46) | 290.7 (283.30, 297.55) | 289.5 (284.16, 297.36) | 1.000^ |
| **FIO2 requirement during ICU stay within 24hr, Median (IQR)** | 75.0 (60.00, 100.00) | 70.0 (60.00, 100.00) | 80.0 (60.00, 95.00) | 0.442^ | 80.0 (60.00, 95.00) | 74.0 (60.00, 100.00) | 80.0 (60.00, 95.00) | 0.971^ |
| **PaO2/FiO2 ratio within 24 hours of admission, Median (IQR)** | 75.0 (50.75, 115.00) | 74.0 (49.00, 116.67) | 79.0 (59.90, 96.40) | 0.321^ | 78.0 (58.89, 104.29) | 77.1 (55.00, 105.78) | 79.4 (59.90, 99.80) | 0.691^ |
| **Heart rate (HR) Baseline (BPM), Median (IQR)** | 103.0 (91.00, 115.00) | 104.0 (92.00, 116.00) | 99.0 (88.50, 110.00) | 0.025^ | 100.5 (89.00, 111.00) | 101.0 (88.00, 113.00) | 99.0 (89.00, 111.00) | 0.991^ |
| **Lowest MAP Baseline (mmhg), Median (IQR)** | 72.0 (63.00, 83.00) | 73.0 (63.00, 83.00) | 72.0 (64.00, 81.00) | 0.378^ | 72.0 (64.00, 80.00) | 71.0 (63.00, 79.00) | 72.0 (65.00, 81.00) | 0.703^ |
| **respiratory rate (RR) Baseline (Breath per minute), Median (IQR)** | 29.0 (24.00, 35.00) | 30.0 (24.00, 35.00) | 27.0 (24.00, 34.00) | 0.503^ | 30.0 (24.00, 34.00) | 30.0 (25.00, 34.00) | 28.0 (24.00, 34.00) | 0.308^ |
| **Maximum temprature Baseline (C°), Median (IQR)** | 37.4 (37.00, 38.10) | 37.5 (37.00, 38.20) | 37.3 (37.00, 37.90) | 0.109^ | 37.3 (37.00, 37.90) | 37.3 (37.00, 37.90) | 37.3 (37.00, 37.90) | 0.972^ |
| *T Test / ^ Wilcoxon rank sum test is used to calculate the P-value.  ^^ Chi square/ ** Fisher’s Exact teat is used to calculate P-value. | | | | | | | | |
