# Supplementary material for: Evaluation of thiamine as adjunctive therapy in COVID-19 critically ill patients: a two-center propensity score matched study
Source: Crit Care. 2021 Jun 30;25:223. doi: 10.1186/s13054-021-03648-9 (PMC8242279; doi:10.1186/s13054-021-03648-9)
Supplement: Supplementary file 2 — Additional file 2: Table 2. Co-existing illness [file 13054_2021_3648_MOESM2_ESM.docx]

**Table e2, Supplementary material, Co-existing illness**

|  | **Before Propensity Score Matching** | | | | **After Propensity Score Matching** | | | |
| --- | --- | --- | --- | --- | --- | --- | --- | --- |
| **Co-existing illness** | **Overall (738)** | **Control (N=650)** | **Thiamine  (N=88)** | **P-value** | **Overall (166)** | **Control (N=83)** | **Thiamine  (N=83)** | **P-value** |
| **Dyslipidemia (DLP), n (%)** | 168 ( 23.2 ) | 144 ( 22.6 ) | 24 ( 27.3 ) | 0.3308^^ | 46 ( 27.7 ) | 25 ( 30.1 ) | 21 ( 25.3 ) | 0.4879^^ |
| **Diabetes mellitus (DM), n (%)** | 442 ( 61.0 ) | 392 ( 61.5 ) | 50 ( 56.8 ) | 0.3949^^ | 92 ( 55.4 ) | 45 ( 54.2 ) | 47 ( 56.6 ) | 0.7548^^ |
| **Hypertension (HTN), n (%)** | 412 ( 56.8 ) | 367 ( 57.6 ) | 45 ( 51.1 ) | 0.2502^^ | 85 ( 51.2 ) | 44 ( 53.0 ) | 41 ( 49.4 ) | 0.6413^^ |
| **Acute Coronary Syndrome (ACS), n (%)** | 12 ( 1.7 ) | 11 ( 1.7 ) | 1 ( 1.1 ) | >0.9999** | 4 ( 2.4 ) | 3 ( 3.7 ) | 1 ( 1.2 ) | 0.3674** |
| **Asthma, n (%)** | 62 ( 8.6 ) | 54 ( 8.5 ) | 8 ( 9.1 ) | 0.8538^^ | 16 ( 9.7 ) | 9 ( 11.0 ) | 7 ( 8.4 ) | 0.5812^^ |
| **Atrial fibrillation ( AFib or AF) , n (%)** | 20 ( 2.8 ) | 18 ( 2.8 ) | 2 ( 2.3 ) | >0.9999** | 6 ( 3.6 ) | 4 ( 4.9 ) | 2 ( 2.4 ) | 0.4430** |
| **Chronic obstructive pulmonary disease (COPD) , n (%)** | 15 ( 2.1 ) | 12 ( 1.9 ) | 3 ( 3.4 ) | 0.4120** | 6 ( 3.6 ) | 3 ( 3.7 ) | 3 ( 3.6 ) | >0.9999** |
| **Cancer , n (%)** | 26 ( 3.6 ) | 26 ( 4.1 ) | 0 ( 0.0 ) | 0.0618** | 2 ( 1.2 ) | 2 ( 2.5 ) | 0 ( 0.0 ) | 0.2393** |
| **Chronic kidney disease (CKD)- (Non-Dialysis), n (%)** | 53 ( 7.3 ) | 48 ( 7.6 ) | 5 ( 5.7 ) | 0.5538** | 13 ( 7.9 ) | 9 ( 11.0 ) | 4 ( 4.8 ) | 0.2090** |
| **Chronic kidney disease (CKD)- (On Dialysis) n (%)** | 25 ( 3.5 ) | 24 ( 3.8 ) | 1 ( 1.1 ) | 0.5538** | 4 ( 2.4 ) | 3 ( 3.7 ) | 1 ( 1.2 ) | 0.2090** |
| **Coronary artery bypass grafting (CABG),n (%)** | 21 ( 2.9 ) | 18 ( 2.8 ) | 3 ( 3.4 ) | 0.7334** | 6 ( 3.6 ) | 3 ( 3.7 ) | 3 ( 3.6 ) | >0.9999** |
| **Heart failure (HF) n (%)** | 59 ( 8.2 ) | 52 ( 8.2 ) | 7 ( 8.0 ) | 0.9400^^ | 11 ( 6.7 ) | 5 ( 6.1 ) | 6 ( 7.2 ) | 0.7708^^ |
| **Hypothyroidism, , n (%)** | 44 ( 6.1 ) | 38 ( 6.0 ) | 6 ( 6.8 ) | 0.7591^^ | 12 ( 7.3 ) | 7 ( 8.5 ) | 5 ( 6.0 ) | 0.5344^^ |
| **Ischemic heart disease (IHD) , n (%)** | 63 ( 8.7 ) | 57 ( 9.0 ) | 6 ( 6.8 ) | 0.4961^^ | 15 ( 9.1 ) | 10 ( 12.2 ) | 5 ( 6.0 ) | 0.1680^^ |
| **Left ventricular clot, n (%)** | 1 ( 0.1 ) | 0 ( 0.0 ) | 1 ( 1.1 ) | 0.1219** | 1 ( 0.6 ) | 0 ( 0.0 ) | 1 ( 1.2 ) | >0.9999** |
| **Liver disease (any type), n (%)** | 15 ( 2.1 ) | 13 ( 2.1 ) | 2 ( 2.3 ) | 0.7035** | 6 ( 3.6 ) | 4 ( 4.9 ) | 2 ( 2.4 ) | 0.4430** |
| **Tuberculosis, n (%)** | 4 ( 0.6 ) | 3 ( 0.5 ) | 1 ( 1.1 ) | 0.4066** | 1 ( 0.6 ) | 0 ( 0.0 ) | 1 ( 1.2 ) | >0.9999** |
| **Venous thromboembolism (VTE) (PE, DVT) , n (%)** | 9 ( 1.2 ) | 8 ( 1.3 ) | 1 ( 1.1 ) | >0.9999** | 1 ( 0.6 ) | 0 ( 0.0 ) | 1 ( 1.2 ) | >0.9999** |
| *T Test / ^ Wilcoxon rank sum test is used to calculate the P-value.  ^^ Chi square/ ** Fisher’s Exact teat is used to calculate P-value. | | | | | | | | |
